# The Lasting Effects of COVID-19 on the Progression of Metabolic Dysfunction-Associated Steatotic Liver Disease (MASLD)

**DOI:** 10.7759/cureus.45231

**Published:** 2023-09-14

**Authors:** Sean Backer, Deepesh Khanna

**Affiliations:** 1 Foundational Sciences, Nova Southeastern University Dr. Kiran C. Patel College of Osteopathic Medicine, Clearwater, USA

**Keywords:** nafld pathogenesis, mash, masld, covid-19 + dili, covid-19 + gut microbiota, hepatobiliary congestion, nash, nafld, non-alcoholic fatty liver disease, covid-19

## Abstract

It is estimated that around 30% of the population living in Western countries has metabolic dysfunction-associated steatotic liver disease (MASLD), a spectrum of pathology (not attributed to alcohol/substance intake) initiated by steatosis and progression toward inflammation and irreversible fibrosis metabolic dysfunction-associated steatohepatitis (MASH). With inflammation being a key component of the transition to MASH, it raises the question of whether the ongoing COVID-19 pandemic, which has notoriously induced hyperinflammatory states, may influence the progression of MASLD. Specifically, it remains unclear if the potential chronic sequelae of COVID-19 in patients who recovered from it may increase the predisposition for MASH. Since MASH maintains a high risk for hepatocellular carcinoma, liver failure, and the need for a liver transplant, the potential additive effects of COVID-19 could prove critical to study. Thus, the objective of this study was to conduct a literature review to examine if COVID-19 could have chronic sequelae that affect the progression of MASLD pathogenesis. It was hypothesized that severe cases of COVID-19 could induce systemic inflammation, metabolic changes, and lasting gut microbiome alterations that lead to inflammatory and fibrotic changes in the liver, similar to those seen in MASH. A scoping review of the literature was conducted utilizing the PubMed database. Studies that examined hepatobiliary pathology, gut microbiome, systemic inflammation, metabolic changes, drug-induced liver injury (DILI), and hypoxia seen in COVID-19 were included. Human studies of adult cohorts, animal models, and in vitro experiments were included. Genetic components of MASLD were not examined. Exclusion criteria also encompassed any studies not referencing the hepatobiliary, gastrointestinal tract, portal system, or systemic circulation. Findings indicated a frequent trend of elevated liver enzymes, mild steatosis, Kupffer cell hyperplasia, and hepatobiliary congestion. It was found that direct cytopathic effects on hepatocytes were unlikely, but the direct viral insult of cholangiocytes was a potential complication. High serum levels of IL-1, TNF-a, and MCP-1, in COVID-19 were found as potential risk factors for MASH development. Hypoxia, altered lipid metabolism, and iatrogenic DILI were also proposed as potential precipitators of MASH development. Notably, lasting changes in gut microbiome were also frequently observed and correlated closely with those seen in MASH.

## Introduction and background

COVID-19 was declared a pandemic in March 2020 and has infected millions of people worldwide [1,2]. The disease manifested a wide range of clinical symptoms involving several organ systems [3-6]. The liver is the second most commonly affected organ in COVID-19, following the lungs [7-9]. Elevations in liver enzymes have been observed in a large proportion of COVID-19 patients and appear to correlate with disease severity [7]. The COVID-19-induced liver damage is believed to be multifactorial in nature and contributed to both indirect (predominantly) and direct insults from the viral infection [7,10,11]. Nevertheless, information on the chronic sequelae of liver damage post-recovery is scarce. Specifically, patients with comorbid metabolic dysfunction-associated steatotic liver disease (MASLD) make up a major risk group for severe COVID-19 illness and may be at immense risk for progression and future complications of their liver condition [10,12]. MASLD is currently the most prevalent liver disease globally, with a prevalence of around 25% [13,14], making it a large risk group for COVID-19 comorbidity [10]. It is estimated that around 30% of the population living in Western countries has MASLD, a spectrum of pathology (not attributed to alcohol/substance intake) initiated by steatosis and progression toward inflammation and irreversible fibrosis metabolic dysfunction-associated steatohepatitis (MASH) [10,13,14]. COVID-19 has a strong correlation to obesity and insulin resistance (two common findings in MASLD), and recent research suggests a correlation to MASLD as well [10].

**Figure 1:**
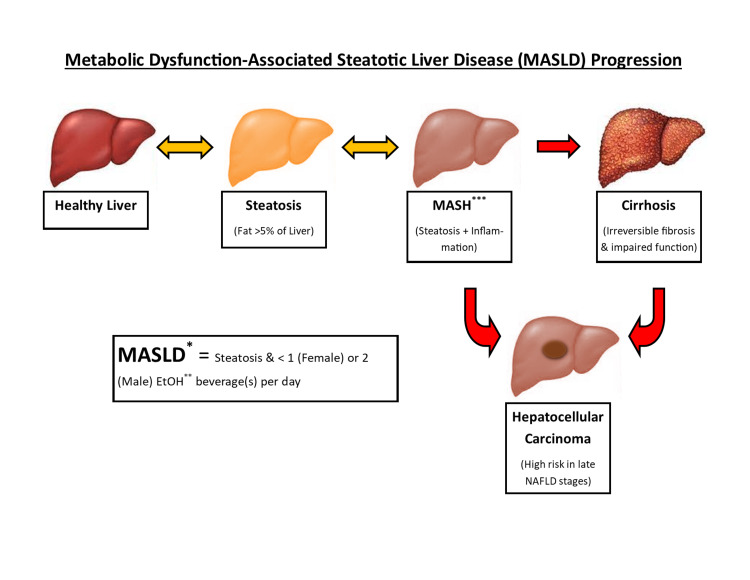
Visually summarized demonstration of the stages of progression of metabolic dysfunction-associated steatotic liver disease. Yellow bidirectional arrows indicate reversible progression, whereas red unidirectional arrows indicate irreversible change. The figure was designed and produced by the authors. Acronyms: * = Metabolic Dysfunction-Associated Steatotic Liver Disease (MASLD); ** = Ethanol (EtOH); *** = Metabolic Dysfunction-Associated Steatohepatitis (MASH).

It is crucial to examine the relationship between COVID-19 infection in MASLD patients, since the prevalence of MASLD is rapidly increasing [14], and any compromise of liver function may affect the metabolism and detoxification of antiviral therapies used in severe COVID-19 infections. MASLD is defined as a progressive spectrum of hepatic pathology (not attributed to alcohol consumption), initiated by reversible steatosis and progressing to non-alcoholic steatohepatitis (NASH) (characterized by inflammation and irreversible fibrosis), until eventually culminating in cirrhosis and liver failure [15,16]. The spectrum of MASLD is visually represented in Figure 1 below. The progression to MASH is seen in around 20% of MASLD patients [14]. However, it remains unclear if this epidemiology could be affected by COVID-19 in the long term. Any effect on MASLD progression by potential chronic liver sequelae of COVID-19, such as inflammation and fibrosis, could affect the incidence of NASH and eventual liver failure in patients who recovered from COVID-19 [15,16]. The chronic effects of COVID-19 on the liver function of MASLD patients are important to study, since exacerbation of the condition may further increase an already prominent incidence of liver failure, hepatocellular carcinoma, and need for liver transplants [11-13]. Thus, more research into chronic sequelae of COVID-19 infections in MASLD patients could prove to save lives and major healthcare expenses in the treatment of hepatic complications. The aim of this literature review is to examine how COVID-19 pathogenesis and pharmacologic treatment can affect the chronic progression of non-alcoholic fatty liver disease (NAFLD).

## Review

Methods and materials

The study was designed as a review of the literature to examine the relationship between the pathophysiological progression of the non-alcoholic fatty liver disease spectrum in relation to COVID-19. Specifically, the objective was to examine the potential lasting effects of COVID-19 pathophysiology and subsequent pharmacologic treatment on the progression of liver pathology in patients with comorbid MASLD.

**Figure 2:**
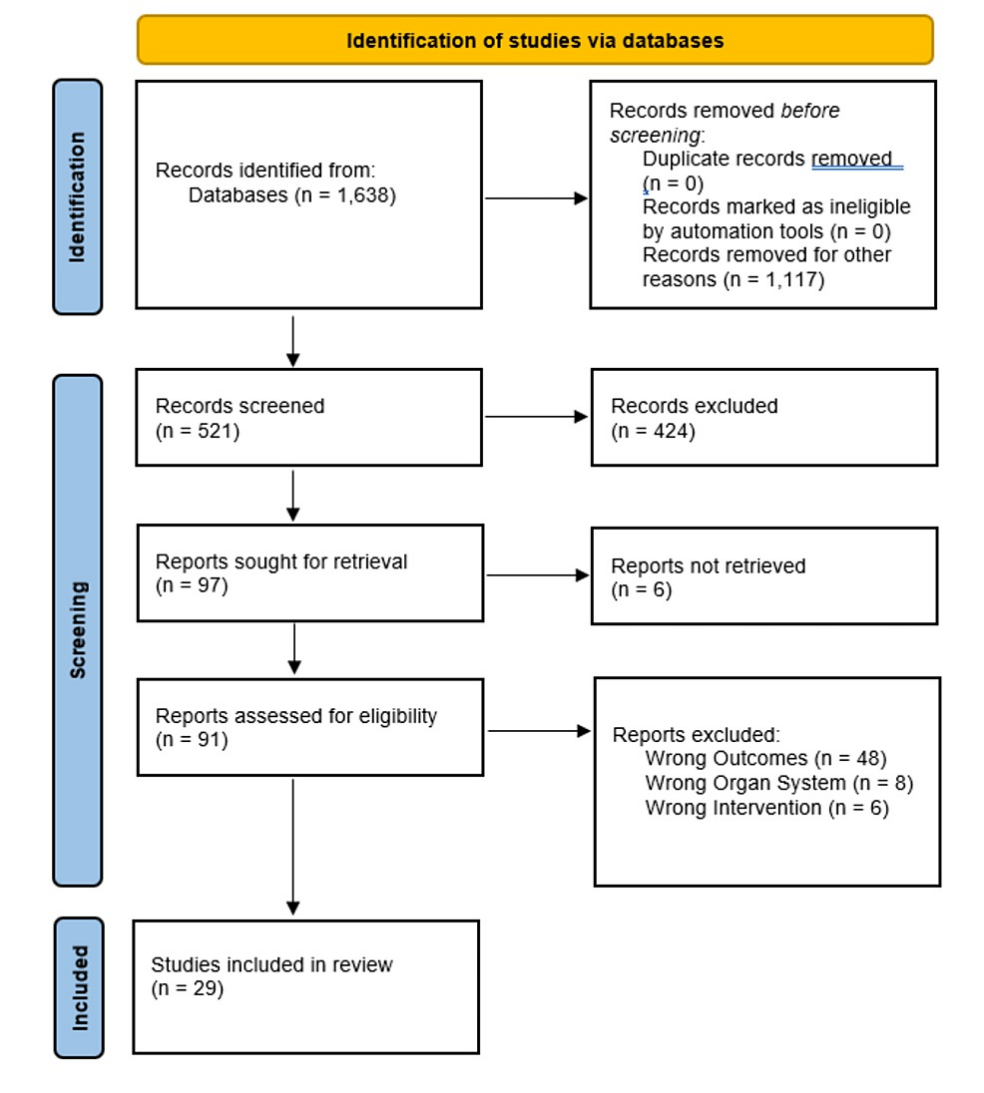
PRISMA 2020 flow diagram of the literature search process in this study. The initial search used the terms mentioned in the methods and materials section for the years 2020-2022 and produced 1,638 results. No automation tools were used for exclusion, but manual exclusion of resources that were books, documents, not full texts, and not free access removed 1,117 results. The resulting 521 articles were screened, and non-pertinent articles were excluded, to eventually yield 29 articles used to meet the objective of this study. The figure was created by the authors, based on the PRISMA 2020 template for literature reviews.

The objective of the study was achieved by performing a database search of PubMed, using the search terms “NAFLD + COVID-19”, “NASH + COVID-19”, “COVID-19 + liver injury”, “COVID-19 + gut microbiota”, and “COVID-19 + DILI” of the years 2020-2022. The initial search yielded 1,638 results. No duplicates were excluded, as PubMed was the only utilized database. A set of initial exclusion criteria was established and narrowed the results down to 521, by excluding abstracts (not full text), full texts that were not free access, and books or documents. Next, a series of inclusion criteria were established to narrow the results down to 97. These inclusion criteria were SARS-CoV-2 infection, hepatic or portal system involvement to some degree, and discussion of pathophysiology. Six articles could not be accessed, which narrowed the search down to 91 articles. The majority of the remaining articles, 48, were excluded as they were irrelevant to the topic, predominantly focusing on the effect of MASLD comorbidity on COVID-19 outcomes (which was the opposite of our study). An additional eight articles were excluded as they predominantly focused on the different organ systems or topics, and six were excluded based on pharmacologic interventions unrelated to COVID-19. A flow diagram, using the Preferred Reporting Items for Systematic Reviews and Meta-Analyses (PRISMA) 2020 template is shown in Figure 2. Aside from the primary results, it should also be noted that another 19 studies were referenced in this review as support of the current pathophysiological models of MASLD progression and pertaining physiological mechanisms.

Pathogenesis of MASLD

**Figure 3:**
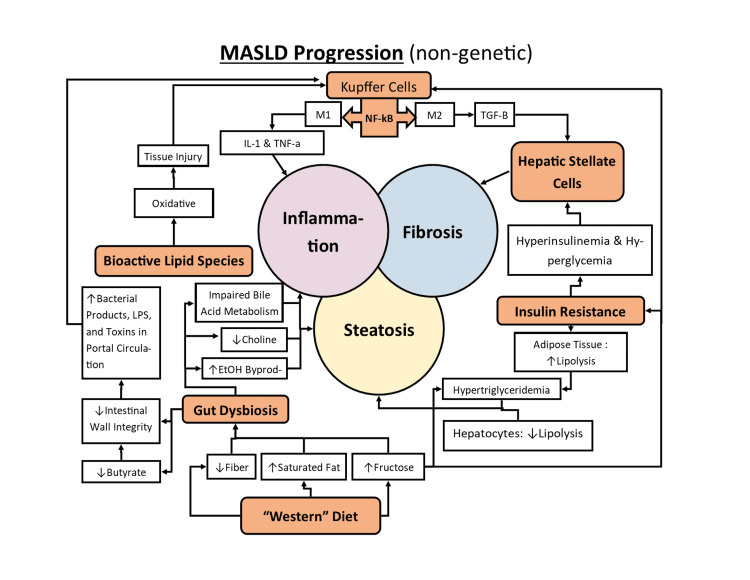
Diagram of the multifactorial pathophysiology of metabolic dysfunction-associated steatotic liver disease. The central circles of steatosis, inflammation, and fibrosis represent the central components of MASLD progression. Note that genetic and epigenetic components have not been represented in this diagram. The figure was designed and produced by the authors. Acronyms: IL-1 = interleukin 1; TNF-a = tumor necrosis factor-alpha; NF-kB = nuclear factor kappa beta; M1 & M2= M1 and M2 Kupffer cell phenotypes; TGF-B = transforming growth factor beta; LPS = lipopolysaccharides; EtOH = ethanol.

MASLD is defined as a spectrum of fatty liver pathology defined by a hepatic fat content greater than 5% and daily alcohol consumption of less than two or one alcoholic beverage(s) in males or females, respectively [15-17]. MASH is defined as the late-stage irreversible form of MASLD (hepatic steatosis), accompanied by hepatic inflammation, hepatocellular ballooning, with or without fibrosis, which may progress to cirrhosis, liver failure, and increase risks of hepatocellular carcinoma [15,16]. The exact pathogenesis of MASLD and MASH remains unclear, yet it is believed to progress according to a multi-hit hypothesis with various environmental and genetic factors [15,16]. These pathological factors can be categorized into an imbalance of lipid metabolism, insulin resistance, gut dysbiosis, genetics, and inflammation [15,16]. The interplay of these numerous factors is visually represented in Figure 3 below. Genetic factors will not be extensively discussed in this review since it is beyond the scope of the study.

Insulin resistance and imbalance of lipid metabolism

As the name suggests, non-alcoholic fatty liver disease is a pathology centered around the deposition of fat in hepatic tissue. The fat deposit predominantly consists of triacylglycerols that are hepatically deposited due to increased lipolysis in adipose tissue, increased triacylglycerol intake from the diet, and hepatic de novo lipogenesis [15,18]. A small cohort study demonstrates the contributions of these processes toward MASLD progression as 59%, 15%, and 26%, respectively [18].

Insulin resistance is a major driver of hepatic fat deposition and is considered a central hallmark of the progression of MASLD [19]. According to a global 2019 meta-analysis, MASLD was found in 55.5% of type 2 diabetes mellitus patients, and MASH prevalence was 37.3% [19]. In a healthy individual, insulin is secreted from pancreatic beta-cells and proceeds to inhibit lipolysis in adipocytes, and in hepatocytes, it induces glycogenesis, inhibits gluconeogenesis, and regulates de novo lipogenesis. The IRS1 and IRS2 hepatic insulin receptors regulate the downstream effects of gluconeogenesis and glycogenesis via the PI3K-PDK-AKT pathway and mediate cell proliferation and survival via the RAS-ERK pathway [20]. However, in the case of hyperglycemia, hyperinsulinemia, and subsequent insulin resistance, the insulin-mediated inhibition of lipolysis in adipocytes is impaired. As a result, triglyceride levels in serum elevate and accumulate in hepatocytes, increasing the liver fat content. Additionally, hepatic insulin resistance, via impaired IRS1/2 sensitivity, will result in impaired glycogen storage, increased gluconeogenesis, and increased triglyceride accumulation in hepatocytes [20]. Excess hepatic triglycerides are also secreted as very low-density lipoprotein into circulation [20]. Overall, insulin resistance induces a state of hypertriglyceridemia and impaired lipid storage by adipocytes, which results in increased lipid accumulation in hepatocytes [15,16].

Eventually, excessive hepatic lipid accumulation results in lipotoxicity via generation of bioactive lipid species, which cause oxidative damage, inflammation, and damage to hepatocytes and ultimately induce fibrosis [21]. Resulting tissue damage can activate Kupffer cells, via transcription factor NF-kB, and induce differentiation to M1 and M2 phenotypes [15,21]. The M1 phenotype is mainly linked to inflammation in MASH, via secretion of pro-inflammatory cytokines TNF-alpha and IL-1 [22]. Meanwhile, the M2 cells are linked to fibrosis, via secretion of TGF-beta and activation of hepatic stellate cells (secrete extracellular matrix) [15,21]. Fibrosis also involves the recruitment of monocytes by Notch-1 activation [20,21].

The impaired downstream signaling of IRS1/2 appears to be a key component of hepatic insulin resistance, yet studies on the correlation of IRS1 and 2 expression to MASLD have yielded contradictory results [20]. However, it does appear that hepatic insulin resistance is also closely linked to inflammation, such as that seen in MASH. Inflammation in MASH has been linked to increased levels of pro-inflammatory cytokines TNF-a and IL-6 [15]. Typically, this hepatic inflammation may arise from NF-kB-mediated Kupffer cell differentiation. Following exposure of pathogenic factors from portal circulation to pattern-recognition receptors (PRR) (such as toll-like receptors and nucleotide oligomerization domain-like receptors), IL-1 may be activated and induce the transcription factor NF-kB. It should be noted that increased pathogenic factors in portal circulation may result from gut dysbiosis in MASLD patients [20]. Nevertheless, these proinflammatory cytokines, NF-kB, and oxidative stress have been shown as factors indirectly inhibiting downstream signaling of IRS1 and IRS2 [20]. The IRS1 and IRS2 inhibition is mediated via JNK, IKK-beta, and SOCS [20]. Thus, the inflammation seen in MASH may further exacerbate hepatic insulin resistance.

Ultimately, the insulin resistance that develops may induce hepatic fibrosis, via indirect and direct activation of hepatic stellate cells (HSC) (main producers of extracellular matrix in MASH). Indirectly, hepatocyte injury due to insulin resistance can activate hepatic stellate cells to produce extracellular matrix (ECM). Macrophage recruitment and subsequent secretion of transforming growth factor-beta (TGF-B) have been implied as the central mechanism of HSC activation in MASH fibrosis [20]. Additional monocytes may be recruited to the liver by CCL2 secretion from Kupffer cells [20]. In stressed hepatocytes, upregulation of transcriptional coactivator PDZ-binding motif (TAZ) and activation of the Notch pathway have also been demonstrated in MASH [20]. Fibronectin may have a protective effect by modulating macrophage differentiation and activity and preventing fibrotic activity [20]. Aside from hepatocyte damage, insulin resistance may also directly induce fibrosis. In hyperinsulinemia, insulin can bind IRS1 and IRS2 on HSCs and induce collagen type 1 production via ERK- and PI3K-dependent signaling pathways [20]. Accompanying hyperglycemia may also aggravate fibrosis via the activation of acid-sensing ion channel 1a on HSCs [20].

Diet and gut microbiome changes

Dysbiosis of the intestinal microbiota has been indicated as an important component in MASLD progression and the incidence of MASH, via decreased intestinal barrier integrity and increased entry of bacterial products and other toxins into portal circulation [15-17]. Bacterial products, such as LPS, may then be recognized by pattern recognition receptors of Kupffer cells and induce differentiation to the M1 and M2 phenotypes that induce inflammation and fibrosis, respectively [21].

A "Western Diet’’ rich in saturated fat and carbohydrates (mainly fructose and sucrose) and low in fiber has been demonstrated as a key promoter of gut dysbiosis, in addition to directly contributing to the insulin resistance and hypertriglyceridemia seen in MASLD [15-17]. Specifically, high intake of fructose (for instance in high-fructose corn syrup) has been strongly linked to MASLD, as it is both an inducer and substrate of hepatic de novo lipogenesis and insulin resistance (discussed previously), in addition to the potential gut dysbiosis [15-17,23,24]. Furthermore, fructose may directly induce hepatic inflammation by activating Kupffer cells via TLR-4 [24]. The reason why fructose may exhibit such strong effects on MASLD progression is likely due to its unregulated metabolism, as it bypasses the rate-limiting step of phosphofructokinase glycolysis [24].

In terms of gut microbiota specifically, a meta-analysis of MASLD patients shows decreased diversity and a phylum level distinguished by a proportional decrease of Bacteroidetes, and an increase of Firmicutes and proteobacteria [14]. The relative abundance of Bacteroidetes is specifically correlated to the progression of MASH [17]. As a result, MASLD/MASH dysbiosis typically manifests as an increased Firmicutes/Bacteroidetes ratio [14]. Additionally, an increase in Streptococcus spp. can be observed in late cirrhotic stages [14].

Various animal studies in mice demonstrate a potential causative relationship of MASLD from gut dysbiosis. Multiple murine studies have shown accelerated development of MASLD and MASH; when germ-free, recipients receive fecal microbiome transplants (FMT) from donors with hyperglycemia and high serum pro-inflammatory cytokines [25,26]. One of the studies utilized two FMT donor groups, one with obesity, hyperglycemia, and pro-inflammatory state, and one donor group with obesity, normoglycemia, and no inflammation. Recipients of the different FMTs produced two distinct phenotypes, with different gut microbiota, suggesting that gut microbiomes from hyperglycemic and pro-inflammatory mice contributed to significantly more steatosis [25]. Another study, where all mice were fed a high-fat diet, showed the presence of hepatic steatosis, necrosis, inflammation, and increased levels of liver enzymes in mice that also received FMT from MASH donors [26].

Pathogenesis of MASLD is also promoted by alteration of the balance of metabolites of the gut microbiota, such as bile acids, choline, short-chain fatty acids, amino acids, and ethanol [17]. In MASLD, the decreased gut microbial diversity infers impaired conversion of primary to secondary bile acids, which promotes increased hepatic inflammation and further disturbance of gut microbiome and bile acid synthesis [17]. The production of the short-chain fatty acid butyrate is also decreased in gut dysbiosis, and thus its properties are lost. Butyrate normally functions as an anti-inflammatory via the activation of regulatory T-cells and inhibition of pro-inflammatory cytokine transcription (via both HDAC-dependent and independent pathways) and promotes the integrity of tight junctions between enterocytes of the intestinal mucosa [17,27]. Thus, low butyrate production, due to gut dysbiosis, may allow entry of LPS and other pro-inflammatory metabolites into hepatic circulation and further promote inflammation seen in MASLD and MASH [17]. Gut bacterial metabolism of aromatic amino acids has also been correlated to MASLD [17]. Lastly, gut dysbiosis may also lead to decreased choline production and increased ethanol production, which may further exacerbate MASLD progression to NASH [17].

SARS-CoV-2 and MASLD pathogenesis progression

As aforementioned, inflammation is an integral component of MASLD progression to MASH and subsequent fibrosis. Insulin resistance, a high fat and high fructose/sucrose diet, and accompanying gut dysbiosis lead to increased inflammation through the portal circulation. However, it is unclear whether systemic hyperinflammatory states, such as that seen in severe COVID-19, may also contribute to the progression of MASLD progression. Previous studies do indicate that systemic inflammatory response syndrome (SIRS), often caused by bacteremia, but also severe COVID-19 may exacerbate both alcoholic liver disease and MASLD [7,21,28].

**Figure 4:**
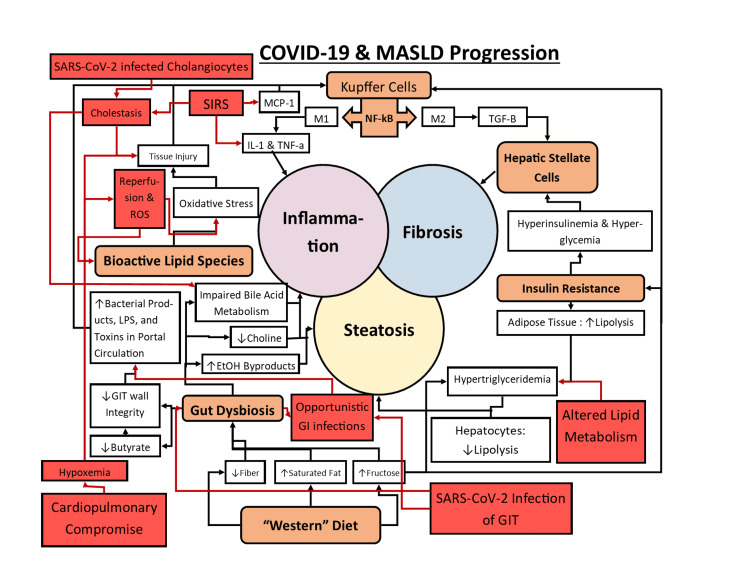
Diagram of the multifactorial pathophysiology of metabolic dysfunction-associated steatotic liver disease with the addition of COVID-19 pathophysiology. Pertinent COVID-19 pathophysiology is demonstrated by red boxes and arrows, to illustrate its effects on MASLD progression. The central circles of steatosis, inflammation, and fibrosis represent the central components of MASLD progression. Note that iatrogenic, pharmacologic, and pathophysiologic factors are not illustrated. The figure was designed and produced by author Sean Backer-Meurke. Acronyms: IL-1 = interleukin 1; TNF-a = tumor necrosis factor alpha; MCP-1 = monocyte chemoattractant protein 1; NF-kB = nuclear factor kappa beta; M1 & M2= M1 and M2 Kupffer cell phenotypes; TGF-B = transforming growth factor beta; LPS = lipopolysaccharides; EtOH = ethanol; GIT = gastrointestinal tract; SIRS = systemic inflammatory response syndrome; ROS = reactive oxygen species; SARS-CoV-2 = severe acute respiratory syndrome coronavirus 2.

Liver damage has been observed in COVID-19 cases in patients both with and without comorbid liver pathologies, especially in severe cases involving acute respiratory distress syndrome (ARDS) [28]. For instance, moderately dysregulated liver-associated enzymes in serum samples of COVID-19 patients have been observed globally and appear to correlate with the severity of illness [7,28-31]. Elevation of transaminases (AST predominant) during hospitalization has been observed in a large proportion of COVID-19 patients, independent of comorbidity, use of statins, muscle damage, and inflammation [7,28,31-33]. A global meta-analysis even concluded that elevation of liver enzymes was significantly correlated with increased severity of illness and mortality, regardless of underlying chronic liver pathology [28]. However, it is unclear what role direct viral insult, indirect sequelae of COVID-19, and potential iatrogenic factors may play in hepatic damage [11,28]. The visual illustration below, in Figure 4, shows the proposed mechanisms of COVID-19 involvement in relation to the pathophysiology of NAFLD.

Direct SARS-CoV-2 damage

It remains unclear if direct infection of hepatic tissue by SARS-CoV-2 has a significant role in NAFLD progression. The virion utilizes the binding of its S-protein to the ACE2 receptor of the host cell to mediate entry [7]. Although the gall bladder and biliary epithelium exhibit high expression of ACE2, the levels are low in hepatic tissues [7,10,34,35]. However, both animal and human studies have demonstrated the upregulation of ACE2 in hepatocytes during inflammation and in fibrotic/cirrhotic conditions of the liver [35]. Inflammation is a characteristic feature of NASH, and fibrosis/cirrhosis often accompanies advanced cases. Thus, a patient with pre-existing NASH may be predisposed to an increased risk of hepatic involvement in COVID-19, thereby exacerbating liver compromise. In addition, in vitro, research has demonstrated an increased ACE2 affinity of beta-corona virus S-protein in the presence of trypsin, which is extensively expressed by liver epithelial cells [11,36,37]. The increased affinity may allow SARS-CoV-2 infection and damage of hepatic tissues, despite lower ACE2 levels, yet this remains hypothetical. Hepatic histopathological studies indicate a non-specific cholestatic pattern of liver pathology without viral cytopathic effects, in addition to enhanced steatosis and Kupffer cell activation [11,28-30,38]. One histopathological study of hepatic biopsies in three living and 25 postmortem COVID-19 patients revealed no signs of SARS-CoV-2 infection or direct insult of hepatic tissues. However, all cases demonstrated Kupffer cell activation, and one-third of cases showed increased steatosis (without underlying comorbidity) [29]. Meta-analysis of COVID-19 liver histopathology studies found Kupffer cell hyperplasia in some cases, but a high prevalence of nonspecific findings, such as hepatic steatosis (55.1%), sinus congestion (34.7%), and vascular thrombosis (29.4%). The metanalysis also was unable to find evidence of any viral cytopathic effects from any primary studies, indicating direct viral infection of hepatocytes as unlikely [30]. In short, it remains unlikely that direct SARS-CoV-2 infection of hepatocytes occurs on any significant scale and that it has any significant implications on the progression of NAFLD (albeit pre-existing steatohepatitis may predispose hepatocytes to SARS-CoV-2 infection). However, nonspecific effects of COVID-19, such as steatosis and Kupffer cell hyperplasia may have real implications for NAFLD progression [11].

Although direct viral insult of hepatocytes appears an unlikely component of COVID-19 infections, cholangiocyte damage is a more likely possibility [32,34]. As previously mentioned, biliary epithelial cells (cholangiocytes) express high levels of ACE2 receptors [10]. Clinical studies have found significant elevations of aminotransferases during hospitalization of between 16-93% of hospitalized COVID-19 patients and appeared to correlate to disease severity, independent of comorbidity, statin use, muscle injury, and inflammation [7,28,31,32]. In addition, elevated gamma-glutamyl transferase levels have been found in as much as 24.4% of hospitalized patients, perhaps indicating viral insult of cholangiocytes [39]. Although histopathological studies examining cholangiocyte damage have been scarce, the use of ex-vivo human cholangiocyte organoids has indicated a significant susceptibility to SARS-CoV-2 infection and an induction of apoptosis via stimulation of CD40, CARD8, and STK4 [32]. The ex-vivo study demonstrates direct viral insult and induction of apoptosis in cholangiocytes and the consequent possibility of bile acid accumulation, which could partially account for elevations in liver enzymes [32]. Elevated bile acid levels have been highly correlated with NASH [40]. Mice models have also indicated increased inflammation and fibrosis when cholestasis is combined with steatosis, relative to steatosis alone [41]. The findings may indicate that NAFLD comorbid COVID-19 patients are increasingly vulnerable to NASH progression secondary to the viral insult of cholangiocytes. However, this hypothesis demands further in-vivo research.

Indirect damage from SARS-CoV-2 infection

Severe COVID-19 cases often lead to SIRS and is characterized by systemic elevations of proinflammatory cytokines, including IL-2, IL-6, IL-7, G-CSF, monocyte chemoattractant protein-1 (MCP-1), macrophage inflammatory protein-1-alpha, and TNF-alpha, with IL-6 noted as a significant contributor to SIRS [10,38]. It should be noted that IL-6 and TNF-alpha are major contributors to the inflammatory progression of NASH [10,15,16]. Moreover, high serum levels of MCP-1, such as those seen in COVID-19, have been shown to exacerbate steatohepatitis [42]. It is believed that the surge of proinflammatory cytokines (specifically IL-6) results from the activation of cytotoxic T-cells and subsequent monocyte activation, following severe infection of the pulmonary epithelium [7]. IL-6 has also been demonstrated to induce cholestasis and act as a cholangiocellular mitogen factor that can lead to inflammatory and fibrotic changes [11]. In combination with potential direct viral insult [32], cholangiocytes may be particularly vulnerable to damage from SIRS secondary to COVID-19 and propagate NASH progression.

Hypoxia, secondary to ARDS (in severe COVID-19), may also cause secondary hepatic damage [7,11,28,33]. Hypoxia can manifest as secondary to cardiopulmonary compromise, as either decreased systolic blood pressure or hepatic venous congestion [7,11]. The notion of hypoxia secondary to hepatic venous congestion can be supported by indicative findings from hepatic histopathology of COVID-19 patient autopsies [29,30]. The hypoxic condition and subsequent reperfusion dysfunction and accumulation of reactive oxygen species can be contributors to hepatocyte damage and inflammation [33], potentially accelerating and mirroring the similar pathogenesis of NASH due to reactive oxygen species secondary to lipid accumulation [21]. Furthermore, hypoxia of fat-laden hepatocytes has been demonstrated to induce activation of pro-inflammatory Kupffer cell phenotypes in both human cell lines and mice [43]. In short, the hypoxic conditions of severe COVID-19 illness appear conducive to the accelerated development of NASH.

Kupffer cells have been implied as a key player in NAFLD progression to NASH. Activation of the transcription factor NF-kB by IL-1 leads to preferred differentiation into the pro-inflammatory M1 over the anti-inflammatory M2 phenotype and upregulation of IL-6 and TNF-a, which are responsible for the progression of inflammation in NASH [15,22]. Extensive activation of Kupffer cells has been observed in the livers of deceased COVID-19 patients, indicating its high level of activity in hyperinflammatory states, a potential shift toward the proinflammatory M1 phenotype, and potential exacerbation of liver damage [11,12,29,30].

Aside from causing a respiratory tract infection, SARS-CoV-2 has commonly been reported to infect the gastrointestinal tract (GIT ) [44-46], with positive stool samples reported in around 50% of cases [45,47,48]. Specifically, biopsy indicates viral tropism and greatest viral load in the glandular epithelium of the gastric, duodenal, and rectal mucosa (all of which exhibit high ACE2 expression) [48]. Additionally, patients with GIT involvement have been shown to have a higher incidence of severe/critical COVID-19, ICU admission, and elevated liver transaminases (AST, ALT) and gamma-glutamyl transferase [44,49]. Interestingly, studies of gut microbiota in COVID-19 patients during hospitalization found decreased microbial diversity (which persisted even after recovery) and increased incidence of opportunistic microbial GIT infections [44,45,47,49]. Metagenomic sequencing of microbiota species was performed longitudinally during hospitalization of a COVID-19 (+) cohort and demonstrated a significant inverse correlation between the abundance of Bacteroides spp. and the severity of COVID-19 [47]. In fact, Bacteroidetes spp. may assert a protective role against SARS-CoV-2 infection by downregulating ACE2 expression [47]. However, decreased Bacteroidetes have not been consistently observed in all studies [49]. Nevertheless, it should be noted that the depletion of Bacteroidetes is also a characteristic finding in the pathogenesis of NAFLD [14, 17]. Furthermore, significant decreases in Clostridia (notably Faecalibacterium prausnitzii) have been observed and may indicate major implications for butyrate metabolism, as they are the main producers of the metabolite [44,47,49,50]. As aforementioned, butyrate supports intestinal wall integrity (tight junctions) and has an integral anti-inflammatory function [17,27]. Thus, decreases in the production of butyrate secondary to gut dysbiosis could predispose to systemic infiltration of microbes from the GIT. Compromised GIT integrity may be particularly relevant in COVID-19 patients, as an increased abundance of opportunistic GIT pathogens was observed [44,45,47]. Since the GIT circulation would immediately drain into the portal system, it raises the suspicion for potential exposure of PAMPs to trigger activation of TLRs on Kupffer cells [21]. This hypothetical pathogenesis may account for the Kupffer cell activation and hyperplasia observed in COVID-19 liver biopsies [11,12,29,30]. Hypothetically, increased activation of Kupffer cells could also predispose hepatic inflammation and fibrosis seen as NASH, especially since persistent gut dysbiosis was observed in recovered COVID-19 patients [47]. Recent studies further support the notion that post-COVID-19 gut dysbiosis may persist long-term [49], with the most recent showing dysbiosis six months post-recovery [51], and could thus continue complicating pathogenesis in NAFLD patients. Additional longitudinal studies on gut microbiota following COVID-19 infections could further examine this hypothesis.

SARS-CoV-2 infection may also alter lipid metabolism, which would already be impaired in NAFLD comorbid patients. The SARS-CoV-2 virus likely alters the lipid metabolism of infected cells as lipids have essential roles in its entry, exit, and replication [52]. Tangibly, alteration in lipid metabolism of COVID-19 patients manifests as decreased total cholesterol, HDL-C, and LDL-C [52,53], and increases in serum triglycerides and VLDL-C [52]. High serum levels of triglycerides (TG) and VLDL-C are both hallmarks of non-alcoholic steatosis [15,16]. Thus, an additional increase in their serum levels during COVID-19 infection could likely accelerate hepatic TG deposition. As NAFLD pathophysiology is linked to insulin resistance and subsequent increased peripheral lipolysis [15,16,20], it is inferable that increased circulation of TG would preferentially accumulate in hepatocytes rather than adipose tissue. The increased serum TG levels could potentially account for the steatosis in histopathology of as many as 55.1% of post-mortem COVID-19 patients [30]. However, there are conflicting results, as a recent meta-analysis found no statistically significant changes in serum TG levels in COVID-19, thus questioning this hypothesis [53]. Nevertheless, the altered lipid metabolism secondary to SARS-CoV-2 Infection could exacerbate hepatic triglyceride deposition seen in NAFLD and thus accelerate disease progression.

Lastly, it is important to note the potential of drug-induced liver injury (DILI) as a result of pharmacological treatment of COVID-19 [7,10,11,38,39]. Guidelines and off-label uses have included antivirals such as ritonavir/lopinavir, remdesivir, antimalarial chloroquine, antibiotics (quinolones and macrolides)(to prevent superimposed bacterial infections), and immunomodulating tocilizumab (monoclonal antibody against IL-6 receptor), which all have potential hepatotoxic effects [7,10,11,33]. The use of corticosteroid therapies may also lead to increased steatosis [11]. A global meta-analysis with 20,874 patients found a total DILI incidence of 25.4% among all COVID-19 patients [28]. Ritonavir/lopinavir and remdesivir were among the agents with the highest incidence of DILI, at 37.2% and 15.2%, respectively [28]. Interestingly, another study found an increased incidence of liver damage with ritonavir/lopinavir, but no correlation with other potentially hepatotoxic drugs [39]. Furthermore, one study found a lower incidence of liver transaminase elevations in COVID-19 patients receiving ritonavir/lopinavir treatment, thus questioning its role in iatrogenic hepatic damage [54]. Theoretically, the monoclonal antibody against IL-6, tocilizumab, could have an anti-inflammatory effect in NASH, as it is effective against hyperinflammatory states of COVID-19, but it has also demonstrated liver enzyme elevations and DILI in a few cases [55]. It should be noted that tocilizumab-induced DILI was reversed in these patients, but it remains unclear if this damage would be reversible in NAFLD comorbid patients [55]. Overall, it appears that iatrogenic liver damage (DILI) can be caused by various pharmacologic treatments and contribute to liver enzyme elevations, thus potentially exacerbating the progression of NAFLD [28]. However, as the data are conflicting, DILI is unlikely to be the only contributor to liver damage in COVID-19 [28,54]. It may be important to monitor liver enzymes in NAFLD patients to prevent further insult [10,28].

## Conclusions

There are numerous components of COVID-19 illness and treatment that have a high potential to contribute to the chronic progression of non-alcoholic fatty liver disease and increase the incidence and severity of NASH. A wide range of studies demonstrate elevation of liver enzymes during COVID-19 hospitalization (correlated to increased severity of the disease), and post-mortem histopathology generally demonstrates a cholestatic pattern of mild liver insult, with frequent presence of steatosis and Kupffer cell hyperplasia, which are all components of NAFLD pathogenesis. Potential long-term progression of NAFLD, following COVID-19 illness, would likely be multifactorial in origin. Indirect effects of SARS-CoV-2 infection, such as hyperinflammation, hypoxia, reperfusion-injury, imbalanced gut microbiome, Kupffer cell activation, and altered lipid metabolism, are all likely to contribute to increased levels of pro-inflammatory cytokines (IL-6, TNF-a, and MCP-1) that are integral in the development of NASH. Adverse effects of pharmacological treatment with potentially hepatotoxic agents are a possible factor in exacerbating NAFLD progression. Careful monitoring of NAFLD comorbid patients receiving these treatments may be necessary in preventing hepatic complications. Direct SARS-CoV-2 insult of hepatocytes has not been extensively supported by evidence and appears unlikely to progress NAFLD severity. However, multiple studies indicate potential cytopathic effects of the virus on cholangiocytes. IL-6 (significantly elevated in SIRS of COVID-19) has also been shown to induce cholestatic effects and inflammatory/fibrotic changes in the biliary epithelium. The number of factors contributing toward potential impairment and insult of the biliary system indicates that this may be an important component of NAFLD progression following COVID-19 illness and should warrant future research. Nevertheless, an increase in proinflammatory cytokines secondary to COVID-19 pathology could be a major contributor to NAFLD progression, but longitudinal studies are necessary to confirm if the transient hyperinflammatory state can lead to chronic progression of NAFLD.

## References

[1] Backer S, Rezene A, Kahar P, Khanna D (2022). Socioeconomic determinants of COVID-19 incidence and mortality in Florida. Cureus.

[2] Hapshy V, Aziz D, Kahar P, Khanna D, Johnson KE, Parmar MS (2021). Covid-19 and pregnancy: risk, symptoms, diagnosis, and treatment. SN Compr Clin Med.

[3] Patel R, Kaki M, Potluri VS, Kahar P, Khanna D (2022). A comprehensive review of SARS-CoV-2 vaccines: Pfizer, Moderna & Johnson & Johnson. Hum Vaccin Immunother.

[4] Moore A, Khanna D (2023). The role of Vitamin C in human immunity and its treatment potential against COVID-19: a review article. Cureus.

[5] Kalia R, Kaila R, Kahar P, Khanna D (2022). Laboratory and point-of-care testing for COVID- 19: a review of recent developments. Cureus.

[6] Patel BM, Khanna D, Khanna S, Hapshy V, Khanna P, Kahar P, Parmar MS (2022). Effects of COVID-19 on pregnant women and newborns: a review. Cureus.

[7] Idalsoaga F, Ayares G, Arab JP, Díaz LA (2021). COVID-19 and indirect liver injury: a narrative synthesis of the evidence. J Clin Transl Hepatol.

[8] Dominquez BC, Hernandez A, Fernandez-Pacheco A, Taylor L, Kahar P, Khanna D (2022). A survey of public health failures during COVID-19. Cureus.

[9] Makhoul E, Aklinski JL, Miller J (2022). A review of COVID-19 in relation to metabolic syndrome: obesity, hypertension, diabetes, and dyslipidemia. Cureus.

[10] Portincasa P, Krawczyk M, Smyk W, Lammert F, Di Ciaula A (2020). COVID-19 and non-alcoholic fatty liver disease: two intersecting pandemics. Eur J Clin Invest.

[11] Nardo AD, Schneeweiss-Gleixner M, Bakail M, Dixon ED, Lax SF, Trauner M (2021). Pathophysiological mechanisms of liver injury in COVID-19. Liver Int.

[12] Ji D, Qin E, Xu J, Zhang D, Cheng G, Wang Y, Lau G (2020). Non-alcoholic fatty liver diseases in patients with COVID-19: a retrospective study. J Hepatol.

[13] Powell EE, Wong VW, Rinella M (2021). Non-alcoholic fatty liver disease. Lancet.

[14] Milosevic I, Vujovic A, Barac A (2019). Gut-Liver axis, gut microbiota, and its modulation in the management of liver diseases: a review of the literature. Int J Mol Sci.

[15] Fang YL, Chen H, Wang CL, Liang L (2018). Pathogenesis of non-alcoholic fatty liver disease in children and adolescence: from "two hit theory" to "multiple hit model". World J Gastroenterol.

[16] Carr RM, Oranu A, Khungar V (2016). Nonalcoholic fatty liver disease: pathophysiology and management. Gastroenterol Clin North Am.

[17] Chen J, Vitetta L (2020). Gut microbiota metabolites in NAFLD pathogenesis and therapeutic implications. Int J Mol Sci.

[18] Donnelly KL, Smith CI, Schwarzenberg SJ, Jessurun J, Boldt MD, Parks EJ (2005). Sources of fatty acids stored in liver and secreted via lipoproteins in patients with nonalcoholic fatty liver disease. J Clin Invest.

[19] Younossi ZM, Golabi P, de Avila L (2019). The global epidemiology of NAFLD and NASH in patients with type 2 diabetes: a systematic review and meta-analysis. J Hepatol.

[20] Fujii H, Kawada N, Japan Study Group of NAFLD (JSG-NAFLD) (2020). The role of insulin resistance and diabetes in nonalcoholic fatty liver disease. Int J Mol Sci.

[21] Gao B, Tsukamoto H (2016). Inflammation in alcoholic and nonalcoholic fatty liver disease: friend or foe?. Gastroenterology.

[22] Lefere S, Tacke F (2019). Macrophages in obesity and non-alcoholic fatty liver disease: crosstalk with metabolism. JHEP Rep.

[23] Rendeiro C, Masnik AM, Mun JG (2015). Fructose decreases physical activity and increases body fat without affecting hippocampal neurogenesis and learning relative to an isocaloric glucose diet. Sci Rep.

[24] Jegatheesan P, De Bandt JP (2017). Fructose and NAFLD: the multifaceted aspects of fructose metabolism. Nutrients.

[25] Le Roy T, Llopis M, Lepage P (2013). Intestinal microbiota determines development of non-alcoholic fatty liver disease in mice. Gut.

[26] Chiu CC, Ching YH, Li YP (2017). Nonalcoholic fatty liver disease is exacerbated in high-fat diet-fed gnotobiotic mice by colonization with the gut microbiota from patients with nonalcoholic steatohepatitis. Nutrients.

[27] Chen J, Vitetta L (2018). Inflammation-modulating effect of butyrate in the prevention of colon cancer by dietary fiber. Clin Colorectal Cancer.

[28] Kulkarni AV, Kumar P, Tevethia HV (2020). Systematic review with meta-analysis: liver manifestations and outcomes in COVID-19. Aliment Pharmacol Ther.

[29] Fassan M, Mescoli C, Sbaraglia M (2021). Liver histopathology in COVID-19 patients: a mono-institutional series of liver biopsies and autopsy specimens. Pathol Res Pract.

[30] Díaz LA, Idalsoaga F, Cannistra M (2020). High prevalence of hepatic steatosis and vascular thrombosis in COVID-19: a systematic review and meta-analysis of autopsy data. World J Gastroenterol.

[31] Bloom PP, Meyerowitz EA, Reinus Z (2021). Liver biochemistries in hospitalized patients with COVID-19. Hepatology.

[32] Zhao B, Ni C, Gao R (2020). Recapitulation of SARS-CoV-2 infection and cholangiocyte damage with human liver ductal organoids. Protein Cell.

[33] Portincasa P, Krawczyk M, Machill A, Lammert F, Di Ciaula A (2020). Hepatic consequences of COVID-19 infection. Lapping or biting?. Eur J Intern Med.

[34] Mohammed A, Paranji N, Chen PH, Niu B (2021). COVID-19 in chronic liver disease and liver transplantation: a clinical review. J Clin Gastroenterol.

[35] Paizis G, Tikellis C, Cooper ME (2005). Chronic liver injury in rats and humans upregulates the novel enzyme angiotensin converting enzyme 2. Gut.

[36] Letko M, Marzi A, Munster V (2020). Functional assessment of cell entry and receptor usage for SARS-CoV-2 and other lineage B betacoronaviruses. Nat Microbiol.

[37] Xia S, Lan Q, Su S (2020). The role of furin cleavage site in SARS-CoV-2 spike protein-mediated membrane fusion in the presence or absence of trypsin. Signal Transduct Target Ther.

[38] Huang C, Wang Y, Li X (2020). Clinical features of patients infected with 2019 novel coronavirus in Wuhan, China. Lancet.

[39] Cai Q, Huang D, Yu H (2020). COVID-19: abnormal liver function tests. J Hepatol.

[40] Zhou T, Kundu D, Robles-Linares J (2021). Feedback signaling between cholangiopathies, ductular reaction, and non-alcoholic fatty liver disease. Cells.

[41] Lionarons DA, Heger M, van Golen RF (2016). Simple steatosis sensitizes cholestatic rats to liver injury and dysregulates bile salt synthesis and transport. Sci Rep.

[42] Gao QY, Chen YX, Fang JY (2020). 2019 Novel coronavirus infection and gastrointestinal tract. J Dig Dis.

[43] Hernández A, Geng Y, Sepúlveda R (2020). Chemical hypoxia induces pro-inflammatory signals in fat-laden hepatocytes and contributes to cellular crosstalk with Kupffer cells through extracellular vesicles. Biochim Biophys Acta Mol Basis Dis.

[44] de Oliveira GL, Oliveira CN, Pinzan CF, de Salis LV, Cardoso CR (2021). Microbiota modulation of the gut-lung axis in COVID-19. Front Immunol.

[45] Kim HS (2021). Do an altered gut microbiota and an associated leaky gut affect COVID-19 severity?. mBio.

[46] Vodnar DC, Mitrea L, Teleky BE, Szabo K, Călinoiu LF, Nemeş SA, Martău GA (2020). Coronavirus disease (COVID-19) caused by (SARS-CoV-2) infections: a real challenge for human gut microbiota. Front Cell Infect Microbiol.

[47] Zuo T, Zhang F, Lui GC (2020). Alterations in gut microbiota of patients with COVID-19 during time of hospitalization. Gastroenterology.

[48] Xiao F, Tang M, Zheng X, Liu Y, Li X, Shan H (2020). Evidence for gastrointestinal infection of SARS-CoV-2. Gastroenterology.

[49] Yeoh YK, Zuo T, Lui GC (2021). Gut microbiota composition reflects disease severity and dysfunctional immune responses in patients with COVID-19. Gut.

[50] Gou W, Fu Y, Yue L (2021). Gut microbiota, inflammation, and molecular signatures of host response to infection. J Genet Genomics.

[51] Chen Y, Gu S, Chen Y (2022). Six-month follow-up of gut microbiota richness in patients with COVID-19. Gut.

[52] Rezaei A, Neshat S, Heshmat-Ghahdarijani K (2022). Alterations of lipid profile in COVID-19: a narrative review. Curr Probl Cardiol.

[53] Zinellu A, Paliogiannis P, Fois AG, Solidoro P, Carru C, Mangoni AA (2021). Cholesterol and triglyceride concentrations, COVID-19 severity, and mortality: a systematic review and meta-analysis with meta-regression. Front Public Health.

[54] Ye XT, Luo YL, Xia SC (2020). Clinical efficacy of lopinavir/ritonavir in the treatment of coronavirus disease 2019. Eur Rev Med Pharmacol Sci.

[55] Serviddio G, Villani R, Stallone G, Scioscia G, Foschino-Barbaro MP, Lacedonia D (2020). Tocilizumab and liver injury in patients with COVID-19. Therap Adv Gastroenterol.

